# Remodeling the Skeletal Muscle Extracellular Matrix in Older Age—Effects of Acute Exercise Stimuli on Gene Expression

**DOI:** 10.3390/ijms21197089

**Published:** 2020-09-25

**Authors:** Matthias Gumpenberger, Barbara Wessner, Alexandra Graf, Marco V. Narici, Christian Fink, Sepp Braun, Christian Hoser, Anthony J. Blazevich, Robert Csapo

**Affiliations:** 1 Research Unit for Orthopaedic Sports Medicine and Injury Prevention, Private University for Health Sciences, Medical Informatics and Technology, Hall 6060, Austria; matthias.gumpenberger@umit.at (M.G.); c.fink@gelenkpunkt.com (C.F.); s.braun@gelenkpunkt.com (S.B.); c.hoser@gelenkpunkt.com (C.H.); 2 Centre for Sport Science and University Sports, University of Vienna, Vienna 1150, Austria; barbara.wessner@univie.ac.at; 3 Institute for Medical Statistics, CeMSIIS, Medical University of Vienna, Vienna 1090, Austria; alexandra.graf@meduniwien.ac.at; 4 CirMyo Myology Center, Department of Biomedical Sciences, University of Padua, 35131 Padua, Italy; marco.narici@unipd.it; 5 Gelenkpunkt Sports and Joint Surgery, Innsbruck 6020, Austria; 6 Centre for Exercise and Sports Science Research (CESSR), School of Medical and Health Sciences, Edith Cowan University, Joondalup, WA 6027, Australia; a.blazevich@ecu.edu.au

**Keywords:** resistance training, sarcopenia, fibrosis, plyometrics, intramuscular connective tissue

## Abstract

With advancing age, the skeletal muscle extracellular matrix (ECM) undergoes fibrotic changes that may lead to increased muscle stiffness, injury susceptibility and strength loss. This study tested the potential of different exercises to counter these changes by stimulating the activity of genes associated with ECM remodeling. Twenty-six healthy men (66.9 ± 3.9 years) were stratified to two of four groups, performing unilateral (i) conventional resistance exercise, (ii) conventional resistance exercise followed by self-myofascial release (CEBR), (iii) eccentric-only exercise (ECC) or (iv) plyometric jumps (PLY). The non-trained leg served as control. Six hours post-exercise, vastus lateralis muscle biopsy samples were analyzed for the expression of genes associated with ECM collagen synthesis (COL1A1), matrix metallopeptidases (collagen degradation; MMPs) and peptidase inhibitors (TIMP1). Significant between-group differences were found for MMP3, MMP15 and TIMP1, with the greatest responses in MMP3 and TIMP1 seen in CEBR and in MMP15 in ECC. MMP9 (3.24–3.81-fold change) and COL1A1 (1.47–2.40-fold change) were increased in CEBR and PLY, although between-group differences were non-significant. The expression of ECM-related genes is exercise-specific, with CEBR and PLY triggering either earlier or stronger remodeling than other stimuli. Training studies will test whether execution of such exercises may help counter age-associated muscle fibrosis.

## 1. Introduction

The aging process into later adulthood is typically accompanied by a progressive loss of skeletal muscle mass and function, known as ‘sarcopenia’, which is associated with adverse outcomes including limited mobility, frailty and increased mortality [1]. A characteristic feature of sarcopenia is that muscle strength and power decline at a faster rate than muscle mass, leading to a decrease in muscle functional quality [2] This is of great clinical concern since measures of muscle strength and power have been found to predict functional decline [3], recurrent falls and fractures [4] and even all-cause mortality [5]. Against this background, significant attempts have been made to reveal the physiological changes underlying the age-associated loss of skeletal muscle’s capacity to generate force and power. The factors contributing to this loss of muscle quality include, amongst others, reductions in muscle fiber density [6] and in particular of myosin protein density [7], changes in muscle fiber and myosin heavy chain composition [8], muscle denervation [9], impairments in muscle fiber activation, neuromuscular junction transmission [10] and excitation-contraction coupling [11] as well as changes in acto-myosin cross-bridge formation [12].

Nonetheless, a less described but important structure that may change with advancing age is the muscle’s extracellular matrix (ECM). The ECM consists of a mesh of collagenous components as well as a mixture of further macromolecules, including various glycoproteins, proteoglycans and elastin [13]. Its functions in mature muscle are manifold and include not only the mechanical support of the muscle fibers, nerves and blood vessels but also the communication with other cells such as myoblasts [14], fibroblasts [15] or inflammatory cells [16]. Of particular relevance to the adaptations to exercise, the ECM is also involved in the regulation of the muscle’s pool of satellite cells [17], which give rise to myogenic progenitor cells that reside within the ECM surrounding muscle fibers and are involved in ECM remodeling in response to hypertrophic stimuli [15]. While it is not yet known whether this remodeling is a prerequisite to permit myofiber hypertrophy or rather happens consequent to it, it is now clear that the ECM plays a major role in the control of muscle growth, repair and regeneration through its influence on satellite cells. Functionally, the intramuscular connective tissue network, which constitutes the collagenous backbone of the ECM, serves as a medium for force transmission along longitudinal [18] and lateral pathways [19,20]. Moreover, it may influence the capability of muscle fibers to radially expand and thus shorten during contraction [21]. The ECM is therefore of central importance for muscle health and function.

Advancing age is typically characterized by decreased turnover of collagens [22], which results in excessive accumulation of intramuscular connective tissue [23] and a concomitant increase in non-enzymatic cross-linking of collagen fibers through the accumulation of advanced glycation end-products [24]. Animal studies suggest that the resulting fibrotic phenotype comes along with a significant increase in muscle stiffness [25,26], which has been associated with altered mechanotransduction as well as chemotactic and inflammatory responses [27,28]. Also, according to animal models the collagen composition may change, particularly in the basal lamina [29,30]. Alterations in this sensitive region may disturb the muscle stem cell niche, leading to a loss, poorer activation and fibrogenic conversion of satellite cells and, consequently, a reduction of the muscle’s regenerative capacity [27,31,32,33]. Functionally, the changes in muscle ECM are expected to negatively affect force transmission along both longitudinal and lateral pathways [34,35,36]. Together, the current evidence suggests that age-associated ECM remodeling renders the muscle stiffer, weaker and more susceptible to injury. It is, therefore, imperative to develop intervention strategies to mitigate these changes. While different countermeasures, including pharmacological or nutritional interventions, could be tested, physical exercise appears to be the most obvious approach due to its demonstrated potential to counter the loss of muscle mass and function and improve functionality and quality of life in older adults [37].

As compared to the well-described effects on contractile muscle cells, little is known about the consequences of physical training on skeletal muscle ECM in humans. An early study by Suominen et al. [38] found prolyl hydroxylase activity to be increased after 8 weeks of endurance training. The only studies to document the effects of prolonged resistance training on muscle ECM have been performed in rodents and suggest that the deposition of connective tissue in older muscles may be reduced by resistance training [39,40]. Acute studies in young humans indicate that genes associated with both the synthesis and degradation of intramuscular connective tissue may be activated through (particularly eccentric) resistance exercise [41,42,43]. Pilot data acquired by our group, however, suggest that the genetic response to exercise might differ between young and elderly subjects, with the latter showing a reduced expression of genes responsible for the biosynthesis of connective tissue-degrading enzymes [44]. Others have proposed that fascial tissues would be best stimulated by the performance of rapid, low-amplitude stretch-shortening cycles (i.e., bouncing movements) or myofascial release techniques like foam rolling, which are assumed to benefit tissue rehydration [45] but these hypotheses are yet to be explicitly tested. Hence, the practices best suited to induce cell turnover in skeletal muscle ECM and particularly its collagenous components, in older age are not currently known.

With the overarching goal of developing long-term training interventions capable of “rejuvenating” the muscle-based ECM, the aim of the present study was to test and compare the effects of different exercise stimuli on the acute expression of genes associated with remodeling of the intramuscular connective tissue network [like COL1A1, MMP2, MMP9] in the vastus lateralis muscle in older age. For this purpose, we recruited a sample of elderly men who were stratified for lower limb muscle quality and then randomly assigned to two out of four possible exercise groups, with exercises being interspersed by two weeks. Vastus lateralis biopsies were taken six hours after cessation of exercise. Considering the evidence from previous research [42,43] and current exercise training recommendations for fascial connective tissues [45], we expected plyometric exercise to provoke the greatest response of genes such as COL1A1 (encoding the biosynthesis of type I collagen) as well as MMP2 and MMP9 (encoding two matrix metallopeptidases, that is, collagen-degrading enzymes), followed by eccentric exercise, concentric-eccentric exercise assisted by myofascial release techniques (i.e., external, manual pressure) and concentric-eccentric exercise alone.

## 2. Results

### 2.1. Subjects Characteristics

The subjects’ anthropometric characteristics, muscle quality indices and 1-RM strength are shown in Table 1.

### 2.2. Gene Expression

The activities of all genes are descriptively shown by exercise group in Table 2. Statistically significant correlations were found between MMP2 and COL1A1 (*r* = 0.775, *p* < 0.001), MMP9 and COL1A1 (*r* = 0.551, *p* = 0.001), MMP3 and TIMP1 (*r* = 0.512, *p* = 0.002), MMP9 and TIMP1 (*r* = 0.610, *p* < 0.001) and MMP15 and TIMP1 (*r* = −0.392, *p* = 0.022). Consequently, ratios of these genes were calculated and compared between exercise groups.

No statistically significant differences between the four exercise groups were found for MMP2, MMP9, COL1A1 or COL7A1 (all *p* > 0.05), although there was a large range in the mean expression between groups in COL1A1 (0.84 to 2.40-fold changes) and MMP9 (0.75 to 3.81-fold changes). For MMP3, a statistically significant difference was found between PLY and CEBR (*p* = 0.024), with gene expression increasing 5.25-fold following CEBR but slightly decreasing (0.93-fold change) after PLY. Similarly, changes in gene expression were greater in CEBR than CE (0.93-fold change; *p* = 0.025). Since univariable models showed that MMP3 gene expression was significantly more upregulated in stronger subjects (*p* = 0.009), a multivariable model accounting for both exercise group and relative 1-RM was run. This model confirmed the statistical differences in MMP3 mRNA expression between exercise groups (PLY v.s. CEBR: *p* = 0.022; CE v.s. CEBR: *p* = 0.023), however the effect of relative 1-RM failed to reach statistical significance (*p* = 0.197).

A significant difference was also found between ECC and CEBR for MMP15 (*p* = 0.045). Here, a small increase in mRNA level was observed in ECC (1.19-fold change) whilst a moderate decrease was observed in CEBR (0.80-fold change). The effects of potentially confounding factors including baseline 1-RM, maximal and integrated EMG activity, exercise session and the leg tested were all non-significant (*p* > 0.05).

**Figure 1:**
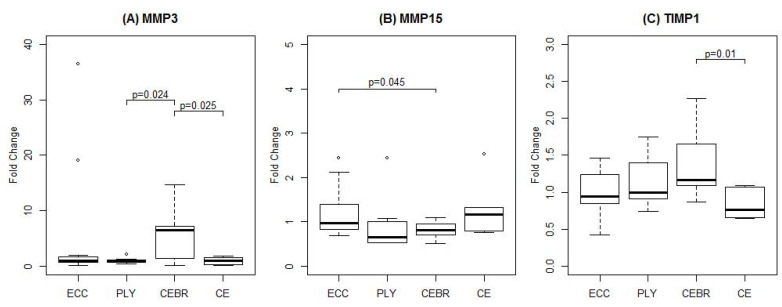
Changes in gene expression six hours after a single bout of unilateral eccentric (ECC, *n* = 13), plyometric (PLY, *n* = 12), concentric-eccentric exercise followed by foam rolling (CEBR, *n* = 12) and conventional concentric-eccentric (CE, *n* = 12) resistance exercise. Fold-changes were calculated with respect to the untrained limb. Brackets indicate significant between-group differences. Outliers, defined as data points located outside 1.5 times the interquartile range above the upper and below the lower quartile, are indicated by open circles.

Changes in TIMP 1 were statistically different between CE (0.83-fold change) and CEBR (1.36-fold change; *p* = 0.010). No significant effects of confounding factors were observed (all *p* > 0.05). Boxplots of all genes for which mRNA levels statistically differed between exercise groups are shown in Figure 1.

### 2.3. Rates of Perceived Exertion

RPE values were 7.67 ± 1.30, 6.75 ± 1.14, 6.31 ± 1.84 and 7.42 ± 1.16 in CE, CEBR, ECC and PLY, respectively. A statistically significant difference was found between CE and ECC (*p* = 0.044), with the perceived exertion being significantly larger in CE. No statistical differences between the other groups were observed. Furthermore, no significant influence of the order of exercise session or the leg tested was found.

## 3. Discussion

The present study compared the acute effects of different exercise stimuli on the mRNA expression levels of genes associated with remodeling of the intramuscular connective tissue network in 24 elderly men. Our results showed that MMP3, MMP15 and TIMP1 were differentially activated by the different exercise modes as well as by foam rolling, with MMP3 and TIMP1 being most strongly stimulated by conventional concentric-eccentric resistance exercise with subsequent foam rolling (CEBR). MMP15, by contrast, was increased the most by eccentric-only training (ECC). Here, we discuss the potential implications of our findings for the development of training interventions that aim to rejuvenate the ECM of skeletal muscles at older age.

The regulation of the ECM and particularly its collagenous components, is a complex process that requires a finely tuned interplay between multiple growth factors, such as transforming growth factor beta or connective tissue growth factor, which may set profibrotic signaling cascades in motion and proteoglycans like decorin and biglycan that act to deactivate these growth factors [47,48]. In addition to collagen biosynthesis regulation, enzymes belonging to the family of matrix metallopeptidases (MMPs) and their antagonists, the tissue inhibitors of matrix metallopeptidases (TIMPs), may influence tissue remodeling by directly degrading various collagen types [49]. The genes examined in the present study (COL1A1, COL7A1, MMP2, MMP3, MMP9, MMP15, TIMP1) were selected based on their demonstrated importance for intramuscular connective tissue remodeling (COL1A1, MMP2, MMP9, TIMP1) as well as on the basis of our pilot study, in which we compared their exercise-induced expression between young and elderly men [44]. The main finding of that study was that conventional resistance exercise (CE) led to a significant upregulation of genes encoding both MMPs (MMP3, MMP9) and collagens (COL1A1) in young men, whereas the same genes were either unaltered (COL1A1) or even downregulated (MMP3, MMP9) in senior men. These observations, together with the notion that aberrant muscle repair of (exercise-induced) muscle damage may be responsible for the fibrotic phenotype often seen in skeletal muscles of older people [50], led us to speculate that standard resistance exercise stimuli, as typically prescribed for older adults [51], may not be ideally suited to stimulate cell turnover in the ECM of older skeletal muscles. The present study was performed in an attempt to identify the most potent exercise stimuli in terms of their capacity to induce renewal of the intramuscular connective tissue network. The exercises tested were selected based on evidence from earlier studies in young individuals that the transcriptional response to a single bout of eccentric contractions was stronger than to concentric contractions [52,53,54] and the partly unsubstantiated, yet frequently purported contention that fascial tissues would react to rhythmic stretch and recoil induced by bouncing movements such as repeated jumps or rehydration practices such as foam rolling [45]. All exercises were perceived as similarly strenuous, with RPE values ranging between 6.3 (ECC) and 7.7 (CE), on average.

COL1A1 is a gene encoding the collagen type I alpha chain, which is a fibril-forming collagen present in all layers of intramuscular connective tissue and the most abundant form of collagen in muscle [55]. Earlier studies performed by us [44] and others [56,57] have shown that an acute bout of conventional (i.e., concentric-eccentric) resistance exercise increases either the expression of COL1A1 or collagen protein fractional synthesis rates within 1.0–8.5 h in young muscle. As compared to these results, the current findings indicate that the exercise-induced activation of genes associated with collagen production may differ between young and elderly subjects, as COL1A1 was decreased 0.84-fold (and COL7A1 virtually unaltered) in CE. Similarly, another study performed in elderly men and women found COL1A1 expression (as measured in the rested state) to be slightly decreased (0.81-fold change) even after 12 weeks of standard resistance training [58]. While care must be taken when extrapolating genetic information to the protein level, the current body of evidence suggests that acute conventional (i.e., concentric-eccentric) resistance exercise triggers a rapid activation of collagen synthesis pathways in young muscle, whereas these anabolic processes may be either delayed or attenuated in older subjects.

Previous studies testing different exercise modalities in young subjects have shown conflicting results. While Holm et al. [56] found muscle connective tissue protein fractional synthesis rates to be significantly more increased in subjects performing eccentric as compared to concentric contractions, Moore et al. [57] reported no respective differences between contraction modes. In our study, which is the first to test the effects of different exercises in elderly humans, substantial between-subject variability precluded the differences in the expression of COL1A1 and COL7A1 between the four exercise modalities tested from reaching statistical significance. Further research in larger samples is required to test the hypothesis that, in elderly subjects, CEBR and PLY (leading to an up-regulation) are more potent in stimulating the expression of genes that encode the biosynthesis of different intramuscular collagens as compared to CE and ECC (leading to a down-regulation).

The activity of collagen-producing pathways, however, must be interpreted in concert with those responsible for collagen degradation. Here, the MMPs 2 (gelatinase A) and 9 (gelatinase B) play a central role in that they are able to hydrolyze type IV collagen, laminin, elastin and fibronectin, that is, components present in the basal lamina. Thus, these MMPs are of crucial importance for the development and repair of muscles after injury, as ECM components must be degraded to allow satellite cells to migrate to and, ultimately, fuse with existing myofibers [59]. In agreement with earlier studies examining their expression in muscle tissue [60], we found MMP2 levels to be virtually unaffected by acute exercise, irrespective of the modality used. Scheede-Bergdahl et al. [61] found levels to be increased after 2 and 8 weeks of rowing exercise in both patients suffering from type 2 diabetes and healthy controls and suggested that prolonged exercise training may be required to increase MMP2 gene expression. As a consequence of the (non-significantly) higher expression of COL1A1 in PLY and CEBR, the MMP2:COL1A1 ratio was lower in these exercise groups, with differences between CEBR (0.80-fold change) and ECC (1.51-fold change) reaching statistical significance. In spite of the lack of significant change in MMP2, it is striking that its expression was strongly and significantly correlated with that of COL1A1 (*r* = 0.78), which underlines the important role that MMP2 might play in controlling collagen biosynthesis. Further studies are required to investigate the temporal kinetics of MMP2:COL1A1 after exercise as well its relationship with collagen I protein synthesis rates.

As opposed to the sluggish response of MMP2, previous studies examining the acute effects of various forms of endurance exercise indicate that the expression of MMP9, which is not only responsible for collagen degradation but also involved in satellite cell activation [62,63] and migration [64], may change more rapidly [65,66,67]. The up to 3.81-fold changes in MMP9 observed in our study confirm the generally high responsiveness of MMP9, although large variability existed both between subjects (reflected by the large SDs shown in Table 3) and exercise modes. In terms of the latter, comparisons between exercise groups showed no statistical differences. Hence, the observed increases in MMP9 and the MMP9:COL1A1 ratio in CEBR and PLY (as compared to no changes or decreases in CE and ECC) warrant further statistical corroboration. The hypothesis that CEBR and PLY may truly trigger a stronger response of genes encoding collagen-degrading enzymes receives some support by our observation that their antagonist TIMP1 was slightly up-regulated in CEBR and PLY (1.14- to 1.36-fold change) and either unaltered or even slightly down-regulated in CE and ECC (0.83- to 0.99-fold change; differences between CEBR and CE statistically significant). It should be noted, however, that TIMP1 reportedly peaks only after completion of myotube formation [64]. Consequently, the collection of muscle samples at 6 h post-exercise may not have allowed TIMP1 levels to reach their maxima.

Statistical differences were found in the exercise-induced expressions of MMP3 and MMP15 genes, which are two less described members of the MMP family. In skeletal muscle, MMP3, also known as stromelysin 1, appears to serve multiple purposes. First, it may be involved in the repair of muscle tissue following injury through the facilitation of cell migration and differentiation and the activation of other MMPs and, possibly, satellite cells [68]. Second, it may act at the neuromuscular junction where it cleaves agrin from the synaptic basal lamina [69]. Agrin is a proteoglycan secreted by motor axons that triggers the aggregation of acetylcholine receptors in the postsynaptic membrane. Thus, it serves to modulate the structure and function of the neuromuscular junction [70] and excessive cleavage has been proposed to promote sarcopenia [71]. Nonetheless, the responsiveness of MMP3 to physical exercise is still poorly understood. Serum levels in men were reported to be either unaffected [72] or increased [73] by resistance exercise, whilst soleus gene expression in mice suffering from peripheral artery disease was reduced nearly 3-fold after chronic endurance training [74]. In our study, a small reduction in MMP3 levels was observed in CE and PLY (both 0.93-fold change), which is smaller in magnitude but directionally similar to that obtained in our pilot study [44]. By contrast, a more than 5-fold increase in MMP3 activity was found after both CEBR and ECC, with differences between PLY and CEBR reaching statistical significance (Table 3). In consequence, the MMP3:TIMP1 ratio was also significantly higher in CEBR (3.49-fold change) than PLY (0.87-fold) and CE (1.06-fold). Further research is required to test the long-term implications of MMP3 activation in terms of remodeling of the neuromuscular junction or other muscular adaptations induced by training.

MMP15 is a membrane-type peptidase that has been associated with angiogenesis and epithelial-mesenchymal transition in tumor progression [75,76]. While its function in muscle is largely unknown, it is interesting to note that its reactions to acute exercise differed from the other MMPs studied in this experiment. Not only was MMP15 the only MMP negatively correlated with TIMP1 (*r* = −0.39), it was also the only MMP downregulated in CEBR and PLY (0.80- to 0.92-fold change) while slightly upregulated in CE and ECC (1.19- to 1.29-fold change; differences between CEBR and ECC statistically significant). As compared to MMP3 and MMP9, however, the absolute exercise-induced changes were of rather small dimension. More basic research is required to elucidate the role of MMP15 in skeletal muscle.

Several limitations of this study warrant consideration. The main purpose of this study was to acquire rapidly available data reflecting the potential of exercises to induce remodeling in the skeletal muscle ECM, to inform their selection for future long-term training interventions. While changes in gene expression may be induced by a single exercise session, the conclusions derived from the data presented here need to be substantiated in histological studies including measures of ECM protein content, structure and composition. Moreover, gene activities were assessed at a single time point only. Thus, the comparisons of exercise stimuli might have been affected by divergent time kinetics of gene expression. The rationale for sampling at 6 h post-exercise was to minimize the effects of any early inflammatory response that may occur between 0–4 h after exercise [77] and to conform with the procedures adopted in our previous study [44]. For comparison of gene activities between exercises, a within-subject study design was used that contrasted the expressions measured in the trained and non-trained limbs. While unilateral training bears the risk of crossover effects arising, for example, from the systemic activity of anabolic hormones like IGF-1, intraindividual comparisons ensure the best possible comparability of both personal (e.g., genetic predisposition, fitness level, …) and environmental (e.g., nutritional status, time of the day, …) factors. Thus, this study design was selected with the intention of increasing statistical power. It must also be noted that muscle biopsy samples contain numerous cell types, including immune cells or fibroblasts, so that altered mRNA levels cannot be directly attributed to muscle fibers. Further, the activities of some genes, such as COL1A1 or MMP9, which are believed to be of major importance for ECM remodeling, were not significantly different between groups although descriptive statistics indicate potential exercise-specific effects. While a total of 96 muscle samples were analyzed for this study and previous investigations reported significant differences in collagen protein fractional synthesis rates between similarly sized groups stimulated with different exercises [56], larger samples need to be studied to increase statistical power. The roles of other genes, for which statistical differences were found (e.g., MMP15), are not yet fully understood in skeletal muscle. Finally, our results do not provide information relating to the specific mechanisms by which the various stimuli might trigger gene expression. Further basic research is required to provide these mechanistic explanations, in particular for the mode of action of foam rolling.

## 4. Materials and Methods

### 4.1. Subjects and Study Design

Twenty-six men (66.9 ± 3.9 years, 1.77 ± 0.05 m, 77.3 ± 8.8 kg) with no history of systematic strength training performed within six months prior to the study volunteered to participate in this study. Subjects suffering from hypertension, heart disease, musculoskeletal restrictions or acute infections were excluded from the study. Taking of anticoagulant but not antiplatelet drugs was also considered a criterion for exclusion. The mini mental state test [78] as well as the mini nutritional assessment [79] were performed to ensure that participants were cognitively capable of understanding the goals and procedures of the study and rule out malnutrition.

**Figure 2:**
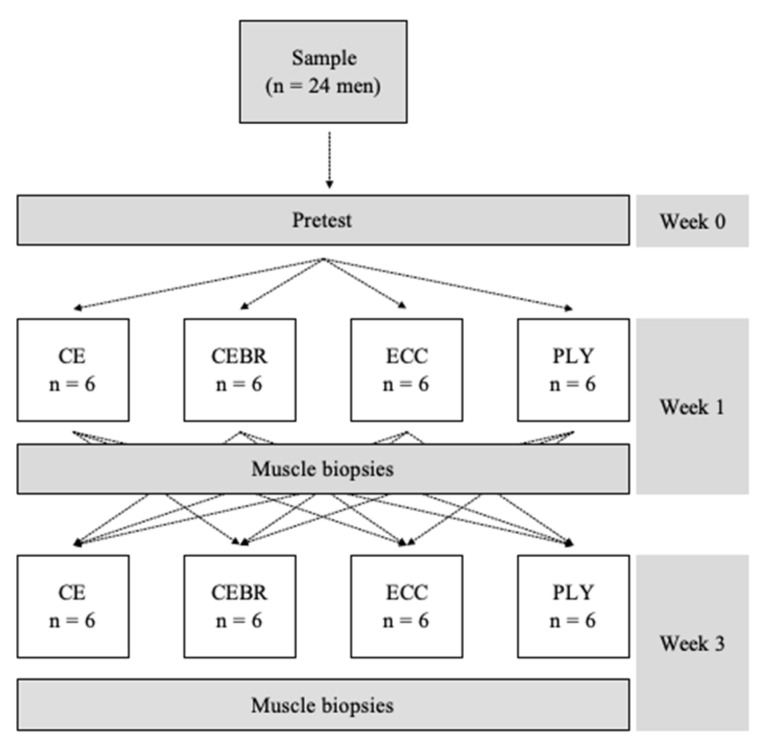
Study design. Vastus lateralis biopsies were taken after completion of the two exercise sessions, which were interspersed by a washout period of two weeks. CE is unilateral concentric-eccentric resistance exercise, CEBR concentric-eccentric resistance exercise followed by self-myofascial release using a blackroll^®^, ECC eccentric-only resistance exercise and PLY plyometric exercise. A standard knee extension device was used for CE, CEBR and ECC. The PLY jumps were performed in a seated position using a commercially available training device.

All subjects were scheduled for three visits. During a pretest, an index of muscle quality was calculated as per the principles described by Barbat-Artigas et al. [46]. The study was carried out using a partial cross-over design (see Figure 2) in which subjects were randomized to two out of the four possible exercise groups [80], including conventional concentric-eccentric resistance exercise (CE), conventional concentric-eccentric resistance exercise followed by self-myofascial release using a blackroll^®^ (CEBR), eccentric-only resistance exercise (ECC) and plyometric exercise (PLY). The randomization was stratified [80] using the index of muscle quality determined in the pretest.

All exercises were performed unilaterally, with the trained leg being changed between exercise sessions and a washout period of two weeks imposed between sessions, two minimize potential crossover effects from the previous exercise. The taking of multiple biopsies from the same muscle is not expected to influence the mRNA response provoked by acute exercise [81]. Three subjects decided to withdraw from the study after the first testing day due to discomfort experienced after the muscle biopsy procedure. Consequently, data were acquired in 12 subjects performing CE, CEBR and PLY and 13 subjects engaging in ECC. Details regarding the exercises tested and the measures taken on each testing day are provided in the following sections.

The study was approved by the ethics committee of the Medical University of Innsbruck (approval code: 1007/2017, approval date: 28 November 2018), *ex ante* registered in the German Clinical Trials Register (DRKS00015750) and conducted in accordance with the Helsinki Declaration. All subjects gave written informed consent.

### 4.2. Pretest

The first session served to (i) administer the mini mental state and mini nutritional assessment questionnaires [78,79], (ii) obtain anthropometric data, (iii) determine the muscle quality index [46] for subject stratification, (iv) measure each subject’s knee extension one-repetition maximum load (1-RM) to set the resistance exercise intensity for the following sessions and (v) familiarize subjects with the proper execution of plyometric jumps on a commercially available jump training device. The muscle quality index was calculated as the ratio of leg muscle power, estimated through a timed sit-to-stand test [82] and total body muscle mass measured by bioimpedance analysis (BIA 101 Anniversary, SMT medical, Germany). To measure the knee extension 1-RM, subjects first performed a short general warm-up on a cycle ergometer (5 min, 1 W∙kg^−1^) and a specific warm-up on a knee extension device (GCEC-340, Body-Solid Inc., Forest Park, IL, USA) against low-to-moderate resistance. From a starting load of 25 kg, the investigator then gradually increased the load until subjects were unable to execute a single repetition with proper form. Sixty s of passive recovery was allowed between trials and care was taken to determine the 1-RM in as few trials as possible (≤ 4), to avoid fatigue-related bias. Subjects then performed ten consecutive bilateral plyometric jumps on the jump training device (Tramp Trainer TT^®^, Frei AG, Kirchzarten, Germany) to familiarize with its usage.

### 4.3. Exercise Sessions

The leg to be trained in the first session was determined by coin toss. Prior to a general warm-up on the cycle ergometer (5 min, 1 W·kg^−1^), bipolar Ag-AgCl EMG electrodes were placed on the vastus lateralis of the trained leg according to SENIAM guidelines [83]. After the general warm-up, three maximal voluntary isometric contractions (MVCs) were performed on the knee extension device, which was fixed at 70° of knee flexion. During MVC execution, EMG data were captured at a sampling frequency of 2 kHz using commercially available software (MyoResearch XP, Noraxon, Scottsdale, AZ, USA). Raw EMG signals were amplified (common mode rejection ratio > 100 dB, input impedance >100 MΩ, gain = 500), band-pass filtered between 10–500 Hz, rectified and then low-pass filtered using a 4th-order Butterworth filter to obtain the linear envelope. The maximal value was extracted and used to normalize the EMG recordings obtained during the subsequent performance of the resistance exercises (CE, CEBR, ECC, PLY). The peak (%MVC) and integrated (%MVC∙s) EMG activities were determined for each set and averaged to quantitatively describe the degree of muscular activation for each exercise. All EMG processing was performed offline using custom-made MATLAB routines (MATLAB R2017b, MathWorks, Natick, MA, USA).

Using a spreadsheet to minimize the between-group differences in the muscle quality index [80], participants were randomly stratified to either one of all four (first session) or one of the remaining three exercises (second session). The following exercise stimuli were tested:

(1) Concentric-eccentric knee extension exercise (CE): Subjects performed 3 × 12 concentric-eccentric repetitions (90° range of motion) at 70% 1-RM with 2 min passive rest between sets. When subjects completed < 12 repetitions in the final set, the exercise was discontinued at the point of full exhaustion. Controlled movement speed and appropriate form were constantly controlled by an experienced examiner.
(2) Concentric-eccentric knee extension exercise followed by foam rolling (CEBR): Subjects performed the knee extension exercise as detailed in a) and then proceeded to roll the lateral, medial, dorsal and ventral aspects of the trained thigh using the foam roller, immediately after EMG electrode removal. All 4 aspects were rolled with 3 × 10 passes, with sets taking 30–40 s to complete.
(3) Eccentric knee extension exercise (ECC): The load for eccentric-only contractions was set at 1.2 × 70% 1-RM. The examiner manually lifted the load and subjects were requested to lower it in a controlled manner. As in groups a) and b), 3 × 12 repetitions were completed with 2 min inter-set rest.
(4) Plyometric exercise (PLY): 3 × 24 single-leg plyometric jumps (2 min inter-set rest) were executed on the Tramp Trainer TT^®^. Subjects were instructed to perform the jumps explosively with accentuated knee flexion during landing to increase the stress placed on the knee extensor muscles. Device inclination was adjusted to 19–21° to ensure that the final set was nearly fully exhaustive.

The subjective rating of perceived exertion (RPE) was recorded after completion of each exercise using a 0 (no effort) to 10 (hardest imaginable effort) scale. The main characteristics of the four exercise stimuli are summarized in Table 3.

### 4.4. Muscle Biopsies

Six hours after completion of exercise sessions, muscle biopsy samples were taken from the middle of vastus lateralis (approximately 50% of the distance between the greater trochanter and the lateral femoral epicondyle) of both legs (trained and untrained). This sampling time point was selected with the intention of minimizing the influence of early inflammatory processes occurring 0 to 4 h after exercise [77] and to conform with the protocol adopted in a pilot study showing a significant, yet age-specific response of the genes analyzed in this study [44]. Five to seven minutes after local anesthesia of the skin with bucaine, the leg was covered with sterile draping. Skin and subcutis were incised over a length of ~5 mm. A 2.1-mm biopsy needle (HistoCore, Bard Biopsy Systems, Tempe, AZ, US) was used on each leg. The needle was inserted twice into the same incision to harvest sufficient tissue. After harvesting, muscle samples were separated from fat, blood and connective tissue before being dispersed in 1 mL Allprotect^®^ Tissue Reagent (QIAGEN GmbH, Hilden, Germany). The samples were stored at 6–7 °C for 24 h and then at −21 °C until further analysis. Total RNA was extracted from about 20 mg of muscle tissue using the RNAeasy^®^ Plus Mini kit (QIAGEN GmbH, Hilden, Germany) according to the manufacturer’s instructions. Samples were disrupted directly in 700 µl of Qiazol provided with the kit using the TissueLyser II (QIAGEN GmbH, Hilden, Germany) operating at 25 Hz for 3 intervals of 2 min. Following chloroform extraction, total RNA was purified using silica-based spin columns. Total RNA was quantified on a spectrophotometer (NanoDrop ND-1000, PeqLab Biotechnologie GmbH, Erlangen, Germany). Purity was checked by calculating 260/280 ratios, which were 1.95 (1.85 – 2.06). RNA (2 µg) was reverse transcribed using the High Capacity RNA-to-cDNA™ kit (Applied Biosystems, Foster City, CA) and stored for further analyses at −40 °C.

### 4.5. Gene Expression Analyses

RNA expression levels were quantified on a 7500 real-time PCR system (Applied Biosystems, Foster City, CA, USA) using standard thermal cycling conditions. Gene-specific primers and probes provided with the commercially available TaqMan^®^ Gene Expression Assays (Applied Biosystems, Foster City, CA, USA) were used to assess the expression of seven genes, which were selected for their demonstrated influence on the intramuscular connective tissue network and the results of a pilot study showing a differential gene expression between young and elderly men [44]: collagen type I alpha 1 chain (COL1A1, Hs00164004_m1), collagen type VII alpha 1 chain (COL7A1, Hs00164310_m1), matrix metallopeptidase 2 (MMP2, Hs01548727_m1), matrix metallopeptidase 3 (MMP3, Hs00968305_m1), matrix metallopeptidase 9 (MMP9, Hs00957562_m1), matrix metallopeptidase 15 (MMP15, Hs00233997_m1) and TIMP metallopeptidase inhibitor 1 (TIMP1, Hs01092512_g1). PCR reactions consisting of the cDNA template (25 ng/well), the gene-specific primers/probe set and the TaqMan^®^ Gene Expression Master Mix (Applied Biosystems, Foster City, CA, USA) were prepared in a total volume of 10 μL according to the manufacturer’s instructions. Each sample was measured in triplicate on 384-well plates. Human skeletal muscle total RNA (AM7982, Fisher Scientific - Austria GmbH, Vienna, Austria) served to prepare the respective standard curves (0.14–100 ng per reaction). Standard thermal cycling conditions were applied using the Quant Studio 7 Flex Real-Time PCR system (Applied Biosystems, Foster City, CA, USA). In order to compensate for variations in the efficiency of the reverse transcription, expression levels were normalized to the average of the housekeeping genes glyceraldehyde-3-phosphate dehydrogenase (GAPDH, Hs02786624_g1) and actin beta (ACTB, Hs01060665_g1), which have been shown to reveal the most consistent C_T_ values among strength training conditions in a previous study [44].

### 4.6. Statistical Analyses

The study was carried out using a partial cross over design. Therefore, for each two-group comparison dependent (for subjects assigned to both compared groups) as well as independent (for subjects assigned to only one of the compared groups) data are available. To investigate the differences in gene expressions between groups and to account for the specific structure of the study design, first univariable mixed model analyses of variance with fixed factor exercise group (CE, CEBR, ECC, PLY) and random factor subject were run separately for each of the gene expression levels (MMP2, MMP3, MMP9, MMP15, COL1A1, COL7A1, TIMP1). Furthermore, to investigate the influence of the potentially confounding factors baseline muscle strength (measured as 1-RM normalized to body mass), maximal and integrated vastus lateralis EMG activity during exercise execution, exercise session (first vs. second) and the leg tested (dominant vs. non-dominant) on gene expression levels, again univariable mixed model ANOVAs (with random factor subject) were run separately for each influencing factor. Then, if the univariable models showed significant effects of more than one factor on the expression of a given gene, multivariable models were performed. In addition to the analyses of single genes, correlations between the expressions of different genes were tested by non-parametric partial correlations using the data acquired in all subjects, accounting for the influence of “subject” (repeated measures due to subjects being assigned to two different exercise groups) [84]. Ratios of pairs of genes found to be significantly correlated were compared between exercise groups using the above procedures. RPE during exercise execution was compared between training groups using linear mixed models accounting for group as factor and subject as random effect. Due to the exploratory character of the study, no correction for multiplicity was done. All *p*-values < 0.05 were considered as statistically significant. The analyses were performed using R, release 3.3.3 (R Development Core Team, Vienna, Austria) and SAS 9.4 (SAS Institute, Cary, NC, USA).

## 5. Conclusions

In conclusion, our study provides data reflecting the effectiveness of different exercise stimuli on the mRNA level of genes associated with skeletal muscle ECM remodeling in elderly men. Six hours after exercise, significant differences were found in the expressions of MMP3, MMP15 and TIMP1, with the greatest responses in MMP3 and TIMP1 seen after conventional resistance exercise followed by foam rolling (CEBR) and MMP15 reacting most strongly to eccentric-only exercise (ECC). Further strong, albeit not statistically significant, upregulations of MMP9, COL1A1 and the MMP9:COL1A1 ratio following CEBR and plyometric training (PLY) suggest that these two exercise modalities trigger either earlier or stronger remodeling of the network of intramuscular connective tissues than the other stimuli tested. Future longitudinal studies will show whether the incorporation of CEBR and PLY into long-term training programs may help counter the fibrotic ECM changes typically seen in older skeletal muscle.

## Figures and Tables

**Table 1:** Anthropometric characteristics, muscle quality and strength by exercise group.

	CE (*n* = 12)	CEBR (*n* = 12)	ECC (*n* = 13)	PLY (*n* = 12)
Age (yr)	67.5 ± 4.7	67 ± 3.3	67.3 ± 4.5	65.4 ± 3.7
Height (cm)	174.5 ± 5.5	176.9 ± 4.6	176.1 ± 4.8	176 ± 4.8
Body mass (kg)	81.5 ± 13	77.4 ± 9.7	77.5 ± 9.9	76.6 ± 8.9
BMI (kg/m^2^)	26.9 ± 4.9	24.7 ± 2.5	24.9 ± 2.5	24.7 ± 2.2
MQI (W/kg muscle mass)	5.3 ± 0.9	5.5 ± 1.1	5.8 ± 1	5.4 ± 0.9
1-RM left (kg)	57.3 ± 8.8	55 ± 5.3	58.6 ± 9.5	54 ± 7.4
1-RM right (kg)	55 ± 7.7	53.5 ± 5.3	57.3 ± 8.8	55.5 ± 6
Rel. 1-RM left (kg/kg body mass)	0.7 ± 0.1	0.7 ± 0.1	0.8 ± 0.1	0.7 ± 0.1
Rel. 1-RM right (kg/kg body mass)	0.7 ± 0.1	0.7 ± 0.1	0.7 ± 0.1	0.7 ± 0.1

CE: Concentric-eccentric knee extension exercise, CEBR: Concentric-eccentric knee extension exercise followed by foam rolling, ECC: Eccentric knee extension exercise, PLY: Plyometric exercise. MQI: muscle quality index [46]. 1-RM: knee extension one-repetition maximum load. Note that all subjects were stratified by the muscle quality index and then assigned to two out of the four exercise groups.

**Table 2:** Expressions of genes by exercise group.

	CE (*n* = 12)	CEBR (*n* = 12)	ECC (*n* = 13)	PLY (*n* = 12)
MMP2	1.00 ± 0.26	0.87 ± 0.28	0.94 ± 0.29	0.89 ± 0.19
MMP3 ^a,b^	0.93 ± 0.69	5.25 ± 4.68	5.41 ± 11.1	0.93 ± 0.52
MMP9	0.75 ± 0.38	3.81 ± 5.66	0.82 ± 0.76	3.24 ± 4.05
MMP15 ^c^	1.29 ± 0.65	0.80 ± 0.20	1.19 ± 0.58	0.92 ± 0.64
COL1A1	0.84 ± 0.36	2.40 ± 4.08	0.88 ± 0.54	1.47 ± 1.45
COL7A1	0.99 ± 0.25	1.07 ± 0.22	1.24 ± 0.53	1.77 ± 2.06
TIMP1 ^b^	0.83 ± 0.20	1.36 ± 0.46	0.99 ± 0.29	1.14 ± 0.34
MMP2:COL1A1 ^c^	1.46 ± 1.02	0.80 ± 0.33	1.51 ± 1.04	0.93 ± 0.51
MMP9:COL1A1	0.95 ± 0.34	2.72 ± 2.61	1.14 ± 1.54	3.31 ± 4.39
MMP3:TIMP1 ^a,b^	1.06 ± 0.79	3.49 ± 2.61	4.98 ± 9.59	0.87 ± 0.57
MMP9:TIMP1	0.90 ± 0.35	3.09 ± 4.98	0.74 ± 0.60	2.69 ± 2.97
MMP15:TIMP1 ^c^	1.68 ± 1.16	0.64 ± 0.26	1.33 ± 0.79	0.98 ± 0.98

CE: Concentric-eccentric knee extension exercise, CEBR: Concentric-eccentric knee extension exercise followed by foam rolling, ECC: Eccentric knee extension exercise, PLY: Plyometric exercise. Values are means ± SD of fold-changes in gene activity in the trained versus untrained limb. The superscript indices ^a–c^ represent significant differences between CEBR-PLY (^a^), CE-CEBR (^b^) and CEBR-ECC (^c^). All subjects were randomly assigned to two of the four exercise groups.

**Table 3:** Characteristics of the exercise stimuli tested.

	Exercise	Sets	Repetitions	Rest	Intensity
1	Eccentric-Concentric Knee Extension	3	12	2′	70% 1-RM ^1^
2	Foam Rolling (after 1)	3	10 (30–40 s)	1’	Pain threshold
3	Eccentric Knee Extension	3	12	1´	70% 1-RM ^1^ × 1.2
4	Plyometric Jumps	3	24	2´	Slope 19–21°

^1^ 1-RM is the concentric knee extension one-repetition maximum load.

## Abbreviations

BIA	Bioimpedance analysis
CE	Concentric-eccentric resistance exercise
CEBR	Concentric-eccentric resistance exercise followed by self-myofascial release through foam rolling using a blackroll^®^
ECC	Eccentric resistance exercise
ECM	Extracellular matrix
MMP	Matrix metallopeptidase
MVC	Maximal voluntary isometric contraction
PLY	Plyometric (jump) exercise
TIMP	Metallopeptidase inhibitor
1-RM	One-repetition maximum load
